# A Data‐Limit Account of Release From Masking During Speech‐on‐Speech Listening

**DOI:** 10.1111/cogs.70166

**Published:** 2026-01-19

**Authors:** Sarah Knight, Yue Zheng, Georgie Maher, Ronan McGarrigle, Sven Mattys

**Affiliations:** ^1^ School of Psychology Newcastle University; ^2^ Department of Psychology University of York; ^3^ School of Psychology University of Leeds

**Keywords:** Speech perception, Masking, Working memory, Attention, Data limit, Individual differences

## Abstract

Speech‐on‐speech listening involves selectively attending to a target talker while ignoring a simultaneous competing talker. Spatially separating the talkers improves performance, a phenomenon known as spatial release from masking (spatial RM). The same is true of spectral separation, that is, filtering the talkers into non‐overlapping frequency bands (spectral RM). The relative benefit of spatial versus spectral RM is currently unknown. Furthermore, it is unclear how listeners’ ability to exploit spatial versus spectral cues is related to individual differences in cognition. The resource‐limit account suggests that cognitive resources are required to support the processing of degraded speech, implying the strongest cognition/performance relationship when RM is limited or absent. However, an alternative claim, referred to as the data‐limit account, suggests that cognitive resources cease to be useful when the target is severely degraded. In this study, participants (*N* = 240) completed a selective listening task in which they transcribed the speech of one of two simultaneously presented talkers. The speech was filtered into interleaved or overlapping frequency bands (spectral RM vs. no spectral RM) and presented dichotically or collocated (a proxy for spatial RM vs. no spatial RM). A battery of cognitive tasks was administered to assess working memory/attention. Spectral RM provided at least as much benefit as spatial RM, with the best performance when both RM types were present. Cognitive scores were significantly positively correlated with RM benefits. However, the weakest correlation between cognitive scores and performance was observed in the no‐RM condition. The results therefore support an account of speech‐on‐speech listening that lies on a continuum from data‐limited to resource‐limited processing as a function of the quality of the target speech signal.

## 1 Introduction

Speech perception in the presence of irrelevant background sound can be difficult for many listeners. Specific challenges are posed by speech‐on‐speech listening, in which the listener must selectively attend to a target talker whilst ignoring a simultaneous competing talker. These challenges arise in part from bottom‐up constraints: access to the target signal is limited by energetic masking (EM)—spectro‐temporal overlap between target and masker, which results in direct competition at the cochlea (Brungart, 2001). Intelligibility depends on how much of the target can be “glimpsed” through spectro‐temporal gaps in the masker (Cooke, 2003); in other words, performance is constrained by the quantity of available data.

However, speech‐on‐speech listening also imposes cognitive demands. Listeners must not only successfully parse the auditory scene into separate streams (segregation) but also allocate attention to the target stream and inhibit the masker (Shinn‐Cunningham, 2008). They may also need to store degraded speech fragments in working memory (WM) for later repair and/or integration into the broader sentence context (Gatehouse, Naylor, & Elberling, 2003). Indeed, selective attention, inhibition, and WM are often seen as closely connected: attentional control is modeled as a key component of WM (e.g., Baddeley, 2000; Engle & Kane, 2004; Unsworth & Spillers, 2010) and good WM is associated with better inhibition of irrelevant sound during selective attention tasks (Conway, Cowan, & Bunting, 2001). Other cognitive resources beyond WM, such as crystallized intelligence, have also been suggested to play a role (e.g., Schneider, Avivi‐Reich, & Danema, 2016; although see Dryden, Allen, Henshaw, & Heinrich, 2017). Both contextual factors and individual differences may affect a listener's ability to draw on this network of relevant cognitive skills (as revealed by, for example, dual‐task paradigms; e.g., Desjardins & Doherty, 2013). Performance will therefore also be constrained by the extent to which cognitive resources are available to process the available data.

### 1.1 Data‐limited versus resource‐limited processes

The distinction between data and resource availability is taken from the work of Norman and Bobrow (1975), as implemented in Mattys, O'Leary, McGarrigle, and Wingfield's (2025) Data‐Resource‐Language (DRL) framework. According to Norman and Bobrow's conception, a task is resource‐limited if performance is determined primarily by the quantity of processing resources allocated to it. However, if performance is broadly independent of the resources allocated, and instead determined primarily by the quantity of data available, then the task is said to be data‐limited. Crucially, the allocation of additional processing resources to data‐limited tasks is unlikely to lead to performance improvements. For example, individual differences in WM skills seem unlikely to affect performance on an audiometric test, which depends primarily on whether the presented tones are above or below listeners’ detection thresholds (as suggested by Heinrich, Ferguson, & Mattys, 2020). In real‐life situations, the majority of tasks are neither fully data‐limited nor fully resource‐limited but lie somewhere along a spectrum. Nevertheless, it is possible to characterize any given task in terms of the relative contributions of the two.

Support for the differential contributions of data and resource limits during speech‐in‐noise listening can be found in a number of studies. For example, Knight, Rakusen, and Mattys (2023) found that cognitive abilities predict speech‐on‐speech listening performance most robustly when EM is reduced or absent. In other words, when data availability is high, performance is more strongly determined by the availability of processing resources than when data availability is lower. Similarly, Janse and Andringa (2021) showed that older adults’ hearing sensitivity was a better predictor of performance for filtered (i.e., perceptually degraded) speech than unfiltered speech, whereas WM capacity showed the opposite pattern. In other words, when data availability is high (unfiltered speech), performance is constrained by the available cognitive resources, whereas when data availability is low (filtered speech), performance is constrained by the amount of additional data that can be gleaned through good hearing acuity. This chimes with the review conducted by Humes (2007), which suggests that the importance of cognition is most pronounced for older adults once audibility is above a given threshold. O'Leary et al. (2023), meanwhile, found that inserting silent pauses into rapid speech improved recall performance when speech was moderately, but not severely, degraded. This finding implies that a data limit was present in the latter case, which prevented additional cognitive processes (i.e., the extra processing time provided by the pauses) from aiding performance. A similar reduction in benefit from silent pauses was also demonstrated for cochlear implant users with poorer, but not better, hearing sensitivity (O'Leary et al., 2023).

The precise position of any given speech‐on‐speech task on the data‐ to resource‐limit spectrum will depend critically on a number of factors. As outlined above, EM—and hence data availability—during speech‐on‐speech listening is determined by spectro‐temporal overlap between target and masker speech. However, speech‐on‐speech listening also requires the ability to break down the acoustic scene into separate streams and selectively direct attention to the target stream. Cues which support this streaming process and subsequent maintenance of attention do not always overlap with cues affecting EM, and they can exert an independent influence on performance. Thus, the position of a speech‐on‐speech task on the data‐ versus resource‐limit spectrum depends on (a) the degree of EM (i.e., glimpsing opportunities) and (b) the presence of streaming cues (such as fundamental frequency differences between the two voices, e.g., Darwin, Brungart, & Simpson, 2003).

When EM is moderate and/or there are acoustic cues available to aid stream segregation, there is some disruption to data availability, but this can be ameliorated by the application of cognitive resources (e.g., Rönnberg, Signoret, Andin, & Holmer, 2022). Within this portion of the data‐ to resource‐limit spectrum, the relationship between cognitive ability and listening performance is positive (i.e., additional cognitive resources result in a performance benefit). The task therefore falls toward the resource‐limit end of the spectrum. However, as EM becomes increasingly high and cues to streaming become less available, it seems reasonable to assume that performance starts to tend toward a data limit: There is simply not enough of the target speech available, or enough reliable streaming cues, for the application of cognitive resources to provide a substantial benefit. At this point, then, one might expect a weaker (or even absent) relationship between cognition and performance. In other words, the task falls toward the data‐limit end of the spectrum.

### 1.2 Release from masking (RM)

One well‐documented way to increase the amount of data available to listeners during speech‐perception‐in‐noise tasks is to spatially separate the target and the masker (Culling & Stone, 2017). As the two are moved apart, there is an increase in the number and duration of spectro‐temporal regions in which the target energy exceeds that of the masker for a given ear (Edmonds & Culling, 2006), thus affording more glimpses. In other words, there is a change in the degree of EM. In addition to this increase in glimpsing potential, strong location‐based streaming cues are also introduced, with the target and masker being reliably associated with different positions in space (Allen, Alais, Shinn‐Cunningham, & Carlile, 2011; Kidd, Mason, & Gallun, 2005). These additional glimpses and enhanced streaming cues can give rise to large improvements in performance, with some studies reporting as much as a 12 dB reduction in the signal‐to‐noise ratio (SNR) required to achieve a set performance level (Freyman et al., 1999)—a phenomenon known as spatial release from masking (spatial RM; Litovsky, 2012).

Similar results have been obtained when a target and masker are presented not just in different real or perceived spatial locations but also when they are presented dichotically over headphones (i.e., with the target and masker presented to separate ears; e.g., Calcus, Agus, Kolinsky, Colin, & Deltenre, 2015; Knight et al., 2023). Although dichotic presentation precludes the kind of central auditory processing involved in true spatial listening—such as the use of interaural time differences (ITDs)—it nevertheless provides both a reduction of EM (to effectively zero at each ear) and strong location‐based cues to streaming (derived from attending to one or the other ear specifically). Such location‐based cues also induce extreme interaural level differences (ILDs), with the signal at a given ear being entirely absent from the opposite ear. In that sense, dichotic RM can be seen as a special case of spatial RM.

RM can also be obtained by separating the target and masker in the spectral domain (spectral RM). As with spatial RM, presenting a target and masker in non‐overlapping frequency bands makes more glimpses of the target data available to the listener and provides an additional cue to streaming, and has been shown to improve speech perception performance compared to when the target and masker are spectrally overlapping (Apoux & Healy, 2010; Kidd, Mason, Best, & Marrone, 2010; O'Neill, Kreft, & Oxenham, 2019). Other types of RM have been shown, such as temporal RM (e.g., Gifford, Dorman, Brown, & Spahr, 2012); however, the focus of this study is on spatial (specifically dichotic) and spectral release.

Little is known about the relationship between dichotic and spectral RM. Specifically, the questions of whether one type of RM is more beneficial than the other, and whether any extra benefit is accrued when both are present, remain open. The underlying mechanisms driving each type of RM and the role of individual differences are also currently unclear. As described above, both dichotic and spectral RM make more of the target data perceptually available by directly reducing EM; they also both provide a clear cue to streaming (location‐ and frequency‐based, respectively). However, dichotic and spectral RM may rely on these two mechanisms (EM reduction vs. streaming) to different degrees. Furthermore, these mechanisms may be underpinned by different cognitive functions, leading to individual differences in the ability to exploit one type of RM compared to the other.

The question of which type of RM is more beneficial may depend on: (a) whether the portions of the signal unmasked through EM reduction provide more data in one case than the other; and (b) whether location or frequency provides a more salient cue for streaming—in other words, whether listeners are more or less able to direct and maintain their attention to a spatial versus a spectral region. Existing empirical work using speech stimuli to address these questions is relatively sparse, but there is some suggestion that listeners rely more heavily on spatial cues than on the spectral cues provided by, for example, talker gender (Allen, Carlile, & Alais, 2008). However, results from a study by Best, Thompson, Mason, and Kidd (2013) using overlapping versus non‐overlapping frequency bands showed robust effects of both spatial and spectral separation. It also appears to be the case that spatial and spectral cues affect different aspects of the segregation and streaming process during speech‐on‐speech listening, and that these effects depend on whether attention is explicitly oriented to the spatial or spectral domain (Ihlefeld & Shinn‐Cunningham, 2008). In short, the picture is mixed.

The question of whether extra benefit is gained when both types of RM are present may again depend on whether one type of RM unmasks additional useful information compared to the other. However, it may also depend on whether streaming is facilitated by the presence of multiple cues compared to a single cue. Best et al. (2013) found the highest performance when both spatial and spectral RM were present. However, this study used concatenated individual words as target sentences and nonsense maskers. Additionally, the spatial separation involved only a lateralization of the masker. The generalizability of these findings to a situation involving naturally produced, meaningful targets and maskers with larger degrees of spatial separation is unclear.

### 1.3 Cognitive contributions to RM effects

In addition to questions about the nature of RM effects per se, it is also unclear how the bottom‐up, perceptual benefits offered by RM interact with listeners’ deployment of cognitive resources. It has been suggested that cognitive resources support speech perception when the signal is degraded (Rönnberg et al., 2022), implying that the relationship between individual differences in cognitive abilities and performance should be strongest when target and masker overlap spatially or spectrally (i.e., when there is no RM). However, the data‐limit account described earlier (cf., Knight et al., 2023; Norman & Bobrow, 1975) suggests that cognitive resources have relatively lower utility when the available target information is severely reduced by the masker, implying that the cognition/performance relationship should in fact be weakest when there is no RM. Furthermore, and as discussed above, the different mechanisms implicated in RM (EM reduction vs. streaming) may recruit different cognitive skills and do so differently for different types of RM. We may therefore see a relatively weak correlation between dichotic and spectral RM. In other words, any given listener may vary in their ability to exploit one type of RM compared to the other. We may also see a different relationship between cognition and each type of RM, with particular cognitive abilities contributing differentially to the ability to exploit dichotic versus spectral cues.

### 1.4 The current study

**Fig. 1:**
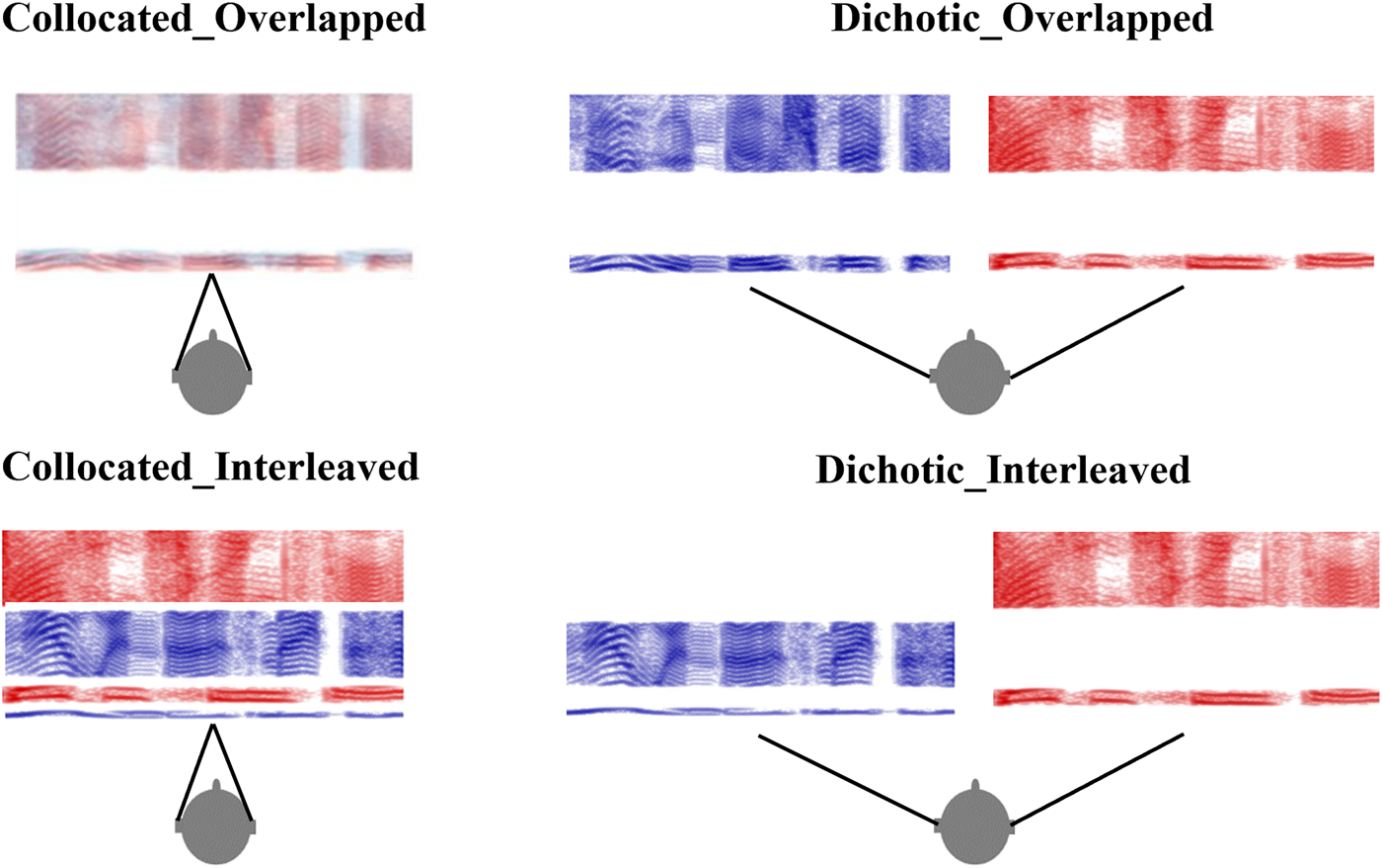
Schematic of the four experimental conditions in the listening task. *Note*. Participants were asked to track one of the two voices. The spectral overlap (overlapped, interleaved) and spatial location of the two voices (collocated, dichotic) were manipulated, leading to listening conditions with no RM, either dichotic or spectral RM, or both types of RM. Note that this figure is a schematic only and does not reflect the relative width of the actual frequency bands used. Please see Table 1 for boundary values of the bands.

In the current study, we addressed these questions via a selective listening paradigm in which participants were asked to track and transcribe the speech of one of two simultaneously presented talkers. On each trial, participants heard a male talker and a female talker simultaneously over headphones. Each talker was uttering a meaningful but low‐predictability sentence (e.g., M: *The box was thrown beside the parked lorry*; F: *Glue the paper to the dark blue background*). Listeners were instructed before the trial to report the sentence spoken by either the male or the female talker. Perceived talker location was manipulated, with the talkers presented via headphones either dichotically or collocated (diotically). The speech was also filtered into frequency bands such that the talkers were either spectrally overlapping or interleaved. There were thus four listening conditions: no RM, dichotic RM only, spectral RM only, or both dichotic and spectral RM (see Fig. 1).

With respect to implementing spatial RM in our experiments, dichotic listening was used as a way of simulating maximal spatial separation. As discussed above, true spatial hearing is best implemented using a combination of ITDs and ILDs, reflecting the cues used during central auditory processing to determine sound source location. Although dichotic presentation does not allow for ITDs, it nevertheless does create ILDs, which have been shown to be a dominant spatial cue at high frequencies and to contribute to the localization of low‐frequency sounds (Middlebrooks & Green, 1991; Middlebrooks & Onsan, 2012). As such, dichotic listening meets our goals of reducing EM and improving streaming through increased perceived left versus right (L‐R) separation. A similar ILD‐only method was used successfully by Knight et al. (2023) to simulate a four‐step change in spatial location using L‐R mixtures with graded intensity differences, and by Calcus et al. (2015), who compared various dichotic listening conditions with a diotic baseline to evaluate spatial masking release. Nevertheless, it must be noted that dichotic listening was used as a proxy for spatial hearing in the current study, and that key differences—particularly with regard to the role of central auditory processing—exist between the two.

To address the contribution of cognitive abilities to performance in general, and to RM benefits in particular, a battery of six cognitive tasks was administered. These tasks were chosen to cover a complementary range of relevant dimensions of WM and attention (e.g., Baddeley, 2000; Diamond, 2013). To assess short‐term phonological memory, we used Non‐word Repetition and Rhyme Judgment tasks, both of which have been used to demonstrate a relationship between speech perception in noise and the ability to manipulate phonological information in WM (e.g., Dryden, 2018; Millman & Mattys, 2017). To assess attention, we used visual versions of Rapid Serial Visual Presentation (RSVP) and Singleton tasks. These two tests were included to reflect the role of visual attention as a reliable predictor of spatial listening (e.g., Clayton et al., 2016). To assess executive function, we used the Reading Span and Letter‐Number Sequencing (LNS) tasks. The former represents a widely used index of complex WM in speech‐in‐noise studies known to tap into both storage and executive control of non‐auditory verbal information (e.g., Rönnberg et al., 2022). The LNS, meanwhile, provides a measure of simple WM, with a greater emphasis on storage and lower demands on executive function, but which has nevertheless been shown to predict speech‐in‐noise performance in a variety of contexts and listener groups (e.g., Knight et al., 2023; Heinrich & Knight, 2016).

Although these tasks were chosen to cover a range of potentially relevant cognitive dimensions, it was unclear which skills might contribute most strongly to performance on our listening task. Each individual cognitive measure also involves task‐specific demands, which are not necessarily relevant to either the cognitive construct under assessment or to speech‐in‐noise perception. Additionally, it was desirable to keep the analyses relatively parsimonious to aid interpretation. We therefore used factor analysis to derive a single cognitive score for each participant from their individual scores on the six tasks, with the aim of reducing task‐specific noise and ensuring the relative contributions of each task were suitably represented.

In terms of listening performance, we expected to see main effects of both spectral and dichotic RM, with performance being higher in the spectrally interleaved than the spectrally overlapping conditions and in the dichotic than in the collocated conditions. If combining both types of RM provides a simple additive benefit, then we would expect the best performance of all in the condition featuring both dichotic and spectral RM but no two‐way interaction. However, it seems plausible to assume that dichotic and spectral RM provide similar benefits in terms of EM reduction—albeit in combination with qualitatively different streaming cues—and thus that we would see only a relatively small extra benefit of adding a second type of RM. If that is the case, we would expect to see a two‐way interaction of dichotic RM × spectral RM, with the improvement in performance resulting from the addition of a second type of RM being somewhat smaller than the improvement resulting from the addition of either dichotic or spectral RM alone.

In terms of the relationship between transcription performance and cognitive abilities, if cognitive abilities are most likely to contribute to speech perception in conditions in which EM is moderate and/or there are cues to streaming, then we expect to see a strong positive relationship between listening task scores and cognitive task scores when one or both types of RM are present. However, when no RM is present—in other words, when EM is severe and there are no cues to streaming—we expected the data limit to attenuate—or perhaps even fully suppress—the contribution of cognition to performance, thus leading to the weakest relationship between cognition and performance in this listening condition.

## 2 Methods

### 2.1 Participants

Participants were recruited via the Human Participant Pool System at the University of York (managed by SONA; www.sona‐systems.com) and the online recruitment platform Prolific (www.prolific.com). All participants provided informed consent before taking part. Testing was conducted via the online experiment builder Gorilla (www.gorilla.sc; Anwyl‐Irvine, Massonnié, Flitton, Kirkham, & Evershed, 2020) between November 8, 2023 and April 24, 2024. Participants recruited through SONA were rewarded with course credit, and participants recruited through Prolific were paid 12 GBP for their time. All participants were self‐reported monolingual native speakers of British English with normal hearing and vision, had no self‐reported language or reading‐related disorders, and were aged between 18 and 35 (mean age = 29 [*SD* = 4.17], female/male = 123/117). Recruitment filters were applied on Prolific so that only people with a Prolific approval rating of 90% and above could access the study. Approval ratings reflect the number of Prolific studies for which a participant has not been rejected by the researcher (e.g., because of attention check failures) and therefore indicate the level of compliance and engagement in previous studies.

Before starting the experiment, participants heard a brief segment of white noise played at the same root‐mean‐square (RMS) level as the stimuli in the main listening task and were instructed to adjust the volume to an audible and comfortable listening level. They then completed a headphone check based on a task designed and validated by Woods, Siegel, Traer, and McDermott (2017). On each trial, participants were asked to identify the quietest of three tones, and the task consisted of six trials in total. The use of antiphase audio for some tones meant that this task could only be completed correctly if participants wore stereo headphones. If a participant responded incorrectly on two or more trials out of six, the headphone check was repeated. If they again responded incorrectly on two or more trials, they were not allowed to proceed to the study.

Power analyses based on pilot studies run in our lab using comparable methodology suggested that the study would require approximately 240 participants (Knight et al., 2023). This sample size should allow detection of both an effect of dichotic/spectral RM (assuming effect sizes equivalent to those in the pilot data) and any significant correlations between our continuous measures (i.e., cognitive task scores) and performance across conditions (assuming correlation coefficients of 0.25, power of 0.90, and alpha levels corrected for multiple comparisons).

### 2.2 Overall procedure

Participants took part in two sessions. Session 1 consisted of the listening task. At least 24 hours after the completion of the listening task, participants completed Session 2. Session 2 consisted of six cognitive tasks, designed to tap into three key components of WM/attention: two phonological short‐term memory tasks (Non‐word Repetition [Phon_NW] and Rhyme Judgment [Phon_RJ]), two attention tasks (Rapid Serial Visual Presentation [Att_RSVP] and Singleton Task [Att_Singleton]), and two executive function tasks (Letter–Number Sequencing [Exec_LNS] and Reading Span [Exec_RSpan]). In total, Session 1 and Session 2 took around 2 hours to complete.

### 2.3 Session 1: Listening task

#### 2.3.1 Materials

The stimuli for the listening task consisted of 184 meaningful sentences drawn from a set of 750 sentences (IEEE sentences; Rothauser et al., 1969) modified to fit modern British English (see Stubbs & Summerfield, 1990). From the selected sentences, 160 were used to form the 80 sentence pairs used in the main listening task, and 24 were used to form 16 practice trials (eight trials with one sentence and eight trials with sentence pairs). All sentences were spoken by the same native male talker and native female talker of British English. The energy spectrum of each sentence was RMS‐equalized to 0.06 Pa and its long‐term average spectrum (LTAS) was matched to the average LTAS of all the sentences before being band‐pass filtered into four non‐overlapping frequency bands (see Table 1). The number of bands and center frequencies were chosen via informal piloting to maximize intelligibility. Each band had a fixed smoothing window of 50 Hz. The bands had logarithmically spaced center frequencies. The upper and lower cut‐off frequencies of the bands were determined via estimations of the equivalent rectangular bandwidths (ERBs): estimations were calculated for center frequencies equivalent to the upper and lower cut‐offs, and cut‐offs were chosen that resulted in no overlap of stimulation on the basilar membrane between the bands after smoothing. The bands were then recombined in pairs to create two types of sentences: those made of odd bands (Bands 1 and 3) and those made of even bands (Bands 2 and 4). The intelligibility of these sentences was verified through pilot testing. Pilot participants (*N* = 21) were recruited and tested via Prolific and Gorilla, and the sentences were presented using the same general procedure as for the main task, including a headphone check. Each participant heard 80 sentences, with counterbalancing ensuring that (a) each sentence was heard in its male and female versions, and in odd bands versus even bands, an equal number of times and (b) each participant heard both an equal number of male and female sentences and an equal number of odd‐band and even‐band sentences. Mean transcription performance for each sentence type (MaleOdd, MaleEven, FemaleOdd, FemaleEven) was 91.95%, 85.75%, 78.50%, and 76.55% respectively. The sentences were then paired to match duration as closely as possible, with each pair consisting of one female and one male talker. The sentences were paired to create two spectral conditions: with spectral overlap (“Overlapped”; i.e., FemaleOdd + MaleOdd or FemaleEven + MaleEven) and without spectral overlap (“Interleaved”; i.e., FemaleOdd + MaleEven or MaleOdd + FemaleEven).

**Table 1:** Lower and upper cut‐offs of the four frequency band filters and their logarithmic center frequencies

Band	Lower Cut‐Off (Hz)	Upper Cut‐Off (Hz)	Lower Cut‐Off With Smoothing Window (Hz)	Upper Cut‐Off With Smoothing Window (Hz)	Centere frequency (Hz)
1	138	218	88	268	173
2	375	713	325	763	517
3	927	2579	877	2629	1546
4	3006	7120	2956	7170	4626

Two versions of each pair were generated to also create two location conditions: collocated (both sentences combined to make mono stimuli played in both channels) and dichotic (each sentence played in a separate stereo channel, one to each ear). Prior to mixing, each band‐pass filtered sentence was RMS‐equalized to the same level (0.06 Pa). Following mixing, all stimuli were once again RMS‐equalized to the same overall level. The mixing process therefore created sentence pairs in four conditions: Collocated_Overlapped, Dichotic_Overlapped, Collocated_Interleaved, and Dichotic_Interleaved.

Fig. 1 presents a schematic illustration of the four conditions. The most challenging condition, Collocated_Overlapped, includes neither spatial nor spectral RM. In contrast, the least challenging condition, Dichotic_Interleaved, includes both spatial and spectral RM. The intermediate conditions have either spatial RM (Dichotic_Overlapped), or spectral RM (Collocated_Interleaved), but not both.

Each target sentence in the listening task contained five keywords, which were used for scoring transcription accuracy expressed as a proportion of keywords correctly transcribed. A typed letter string was scored as correct if it omitted one letter, if it added one letter, if two consecutive letters were transposed (e.g., “lihgt” rather than “light”), or if it was phonetically the same as the keyword (e.g., “lite” rather than “light”). Strings of words in which spaces were omitted were separated if they made up two correct consecutive words in the sentence. Practice trials were excluded from the analysis.

#### 2.3.2 Procedure

Participants first adjusted their volume and completed the headphone check task described above. Participants who passed the headphone check proceeded to the main listening task, in which they heard the sentence pairs described above. Participants were instructed to pay attention to one of the two talkers in each pair and report what the talker said at the end of each trial. They were not told that the sentences contained keywords or how they would be scored. The assignment of target talker (male or female) remained the same throughout the task for each participant and was counterbalanced across participants. Thus, a given participant was asked to report the same voice throughout the experiment. On each trial, participants were presented with a fixation cross on the display screen whilst the audio stimulus played. At the end of the audio stimulus, participants were prompted to type the target sentence into a response box and press the enter key to commence the following trial. They were given a maximum of 60 s to respond before the next trial started.

The listening task consisted of 80 trials, with a break after 40 trials. The task used a two‐by‐two factorial design, with location (collocated/dichotic) and spectral overlap (overlapped/interleaved) as two fully crossed independent variables, creating four conditions of 20 trials each.

All participants heard the same 80 sentence pairs, but the allocation of each unique sentence pair to conditions was counterbalanced across participants. Within each of the four conditions, there were two blocks of 10 trials each, with eight blocks in total. Within each overlapped condition, one block consisted of FemaleOdd + MaleOdd sentences, and the other block consisted of FemaleEven + MaleEven sentences. Within each interleaved condition, one block consisted of FemaleOdd + MaleEven sentences, and the other block consisted of FemaleEven + MaleOdd sentences. The presentation order of blocks, and the presentation order of sentence pairs within each block, were randomized. For each unique sentence pair, the two sentences were presented in all possible combinations of male/female and band manipulation (odd‐ or even‐band filtered) an equal number of times across all participants. Each participant heard an equal number of sentences from male and female talkers, as well as an equal number of sentences filtered by odd and even bands.

The 16 practice trials were presented at various points throughout the experiment. Two practice trials were played prior to the listening task, and two practice trials were played between the blocks of the listening task. For each pair of practice trials, the first trial consisted of the target talker heard in isolation in the same bands (odd or even) as in the block which followed. The second practice trial consisted of a sentence pair with the same band and spatial manipulation as the sentence pairs in the following block. The practice trials ensured that participants had experienced the spatial location and masking configuration of the following block before the block commenced.

### 2.4 Session 2: Cognitive tasks

Session 2 consisted of six cognitive tasks as described in the Overall Procedure section.

#### 2.4.1 Materials

*2.4.1.1. Non‐word Repetition Task (Phon_NW)*: Participants were required to decide if two auditorily presented sequences of monosyllabic non‐words were identical or not. The sequences were presented in two conditions: deviant, where the two sequences differed by one vowel phoneme positioned randomly within the sequence, and non‐deviant, where identical sequences were played. There were 28 trials (14 deviant, 14 non‐deviant), with the sequence increasing by one non‐word every four trials, from a minimum of two to a maximum of eight non‐words per sequence. Within a set of four trials, trial presentation order was randomized across participants. After each trial, participants responded by clicking the button labeled “same” or “different” displayed on their screen with no time limit.

The stimuli were generated from the English Lexicon Project (Balota et al., 2007). The initial search criteria consisted of non‐words with no orthographic neighbors that were three to six characters in length. From these, monosyllabic non‐words that did not bear a strong similarity to an identifiable word or name were selected. The non‐words were created using text‐to‐speech software (the female talker “Amy” from ttsmp3.com). Deviant non‐words were prosodically matched to their non‐deviant (identical) counterparts as far as possible. There was a 500‐ms gap between non‐words within a sequence and a 2000‐ms gap between sequences.

A *d’* score was calculated to measure participants’ ability to discriminate between sequences whilst also accounting for any bias toward choosing either “Same” or “Different.” *d’* was measured using the proportion of “Different” trials that received a “Different” response and the proportion of “Same” trials that received a “Different” response. The *d’* scores were then standardized (*z*‐score) and used as the Phon_NW score in further analyses.

*2.4.1.2. Rhyme Judgment Task (Phon_RJ)*: Participants were instructed to determine as quickly and accurately as possible if two visually presented words rhymed with each other. Word pairs were taken from Johnston and McDermott (1986) and formed four conditions: rhyme/orthographically similar (R+O+), rhyme/orthographically dissimilar (R+O−), does not rhyme/orthographically similar (R−O+), does not rhyme/orthographically dissimilar (R−O−). Potentially unfamiliar words and words with possible individual variability in phonological representation were replaced. All words were four letters long and were presented in monospace font to ensure that word length was not indicative of rhyming status. Participants used their keyboard to respond by pressing the A key for “no” and L key for “yes,” with visual labels on screen to remind participants which button referred to which response. There was no time limit for participants to respond. Following the button press, there was an 800‐ms gap before the next word pair. The trial order was randomized across conditions. There were 92 trials in total (23 trials per condition).

Both response accuracy and reaction time (RT) were measured, but only the R+O− and R−O+ conditions were used in the analyses due to likely ceiling effects in the R+O+ and R−O− trials (Dryden, 2018). Response accuracy was the proportion of correct incongruent trials, and RT was the average RT of correct incongruent trials. Both measures were *z*‐transformed and integrated using the balanced integration score method (BIS; Liesefeld & Jancyzk, 2019). In this way, each participant received a score that took into account speed‐accuracy trade‐offs, which may occur when participants are asked to answer “as quickly and as accurately as possible.”

*2.4.1.3. Rapid Serial Visual Presentation Task (Att_RSVP)*: Participants were presented with two rapid streams of sequentially presented letters on either side of a central fixation cross. In the selective‐attention condition, participants were instructed to fixate on the cross whilst monitoring one of the two streams (indicated by an arrow prior to each trial). In most trials, a number (3, 4, 6, 7, 8, or 9) appeared at some point in one of the streams, and participants indicated whether or not the number was presented on the cued side by pressing the “Y” key for “Yes” or the “N” key for “No.” In the divided‐attention condition, participants were instructed to fixate on the cross whilst monitoring both streams. A number appeared at some point in one of the streams, and participants indicated on which side the number was presented by pressing “A” for left, “L” for right, or spacebar for neither. For both conditions, visual reminders remained on screen to indicate which button referred to which response. Five trials within each condition did not include a number. Participants were informed of this to avoid tactical responding in the divided attention condition (i.e., using the absence of a number in one stream to infer its presence in the other).

The target number appeared in each stream (left/right) an equal number of times in each condition. The position of the number within each sequence was randomized but was never the first or the last in the sequence. There were 10 letters in each letter stream, with each pair of letters (left/right) presented for 50 ms and no gaps between pairs. Letters I, Z, S and numbers 1, 2, 5 were excluded due to visual similarity. Conditions were blocked, but block presentation order was counterbalanced across participants. There were 70 trials in total (35 selective attention, 35 divided attention).

The difference in performance accuracy between the divided and selective conditions was calculated for each participant. This allowed for the cost of dividing attention between two sources of information to be assessed. This difference was *z*‐transformed and multiplied by −1 to ensure larger values reflected better performance as in the other cognitive tasks.

*2.4.1.4. Singleton Task (Att_Singleton)*: Participants were presented with eight outlined circles evenly positioned around the perimeter of a larger imaginary circle. Seven circles contained a letter, and one circle contained a number (1–4), which served as the target. The circles were presented for 1 s. The larger imaginary circle was divided into two halves vertically, with each half containing four of the smaller circles. Participants were instructed to pay attention to only the set of circles on one half of the perimeter (left/right), indicated prior to each trial. The target number was always shown in this half, and participants had to press the corresponding number on their keyboard as quickly as possible when they detected it. In the “with‐singleton” condition, one of the non‐target circles was simultaneously presented in red (i.e., a visual singleton). The visual singleton was always in the opposite half of the circle to the target number, to maximize distraction from the target. In the “without‐singleton” condition, there was no visual singleton present. There were 64 trials (32 per condition), and trial order was randomized across conditions. Each number was presented in each circle twice, once in each condition. For each number, there was a singleton in each of the eight circles once. Letters I and Z were removed due to visual similarity to numbers 1 and 2.

RT was measured as the time between stimulus onset and the participant's keyboard response. The *z*‐transformed difference in RTs between correctly answered with‐singleton and without‐singleton trials was used to score performance. This difference assessed the cost of inhibiting a distractor (i.e., a singleton).

*2.4.1.5. LNS Task (Exec_LNS)*: Participants heard a sequence of numbers and letters spoken by a male talker. After hearing each sequence, they were instructed to type the numbers in ascending order, followed by the letters in alphabetical order (Wechsler, 1997). Sequences began with a length of two items and increased in length by one item every three trials, with a maximum length of eight. Each item was presented 1 s after the onset of the previous item. If all three trials of a particular length were answered incorrectly, the task was terminated.

Each trial was scored as either correct or incorrect. A trial was scored as correct only if there were no discrepancies between the response and the sequence presented. Each participant received a score as the total number of trials correctly responded to (with a maximum of 21). The scores were then *z*‐transformed.

*2.4.1.6. Reading Span Task (Exec_RSpan)*: Participants were required to read sets of written sentences, which were presented one word at a time. Each word was presented for 500 ms, with no gaps between words. Following the presentation of each sentence set, participants were instructed to type the final words of each sentence in the set and respond to a question relating to the semantic content of one sentence within that set. Sentences were taken from the original Daneman and Carpenter (1980) reading span task (RSpan) and modified to remove any outdated or potentially distressing content. There were eight trials in total. After every two trials, the length of the set increased by one sentence, from a minimum of three sentences to a maximum of six sentences per set.

Trials were scored as correct if the participant correctly transcribed all final words of each sentence set. The scoring rules for misspelt words were the same as in the listening task. Performance was calculated as the *z*‐transformed proportion of correct trials. Responses to the semantic questions were recorded but not used in further analysis.

#### 2.4.2 Procedure

Participants were instructed to use the same computer and headphone set‐up as in Session 1. Session 2 began with a simple audio check. First, white noise was played at the same RMS level as in Session 1, and participants adjusted their volume to an audible and comfortable listening level. Following this, three trials of three spoken digits were played, and participants were asked to type these digits in the order they were presented. Before being included in the analysis, participant performance was reviewed to ensure that at least two trials had been answered correctly.

Participants then performed the six cognitive tasks in the following order: (1) Non‐word Repetition Task (Phon_NW); (2) Rhyme Judgment task (Phon_RJ); (3) RSVP Task (Att_RSVP); (4) Singleton Task (Att_Singleton); (5) LNS Task (Exec_LNS); (6) RSpan (Exec_RSpan). Session 2 took around 1 h to complete.

## 3 Analyses

All analyses were conducted using R (version 4.4.0, embedded in R Studio version 2024.04.2) and the packages *dplyr* (1.1.4), *emmeans* (1.10.1), *lme4* (1.1‐35.3) and *psych* (version 2.5.6). To investigate the general effects of spectral and dichotic RM in the listening task, we fitted two generalized linear mixed‐effects models (GLMMs) with trial‐level transcription scores from the listening task as the outcome variable. We modeled each trial as a data point, using a binomial distribution and a logit link. Both GLMMs had spectral separation, dichotic separation, and their interaction as fixed effects. The first GLMM also included the six cognitive task scores in interaction with dichotic separation and spectral separation to assess whether the influence of each individual cognitive ability on performance varied across listening conditions. For the six cognitive tasks, a correlation matrix was generated to explore the strength and direction of the relationship between pairs of tasks and the internal consistency of each task was assessed using Cronbach's alpha. In an attempt to generate a measure of general cognitive ability for each participant, we conducted an exploratory factor analysis on the scores from the six cognitive tasks using the *stats* package (4.4.0) in R. In this analysis, we specified a single underlying factor, which was then used to generate a single score (CogFact) for each participant via the regression method (DiStefano, Zhu, & Mîndrilă, 2009). We then incorporated the individual CogFact scores (*z*‐transformed) into a second GLMM, in interaction with dichotic separation and spectral separation, to assess the influence of general cognitive ability on listening performance.

## 4 Results

**Fig. 2:**
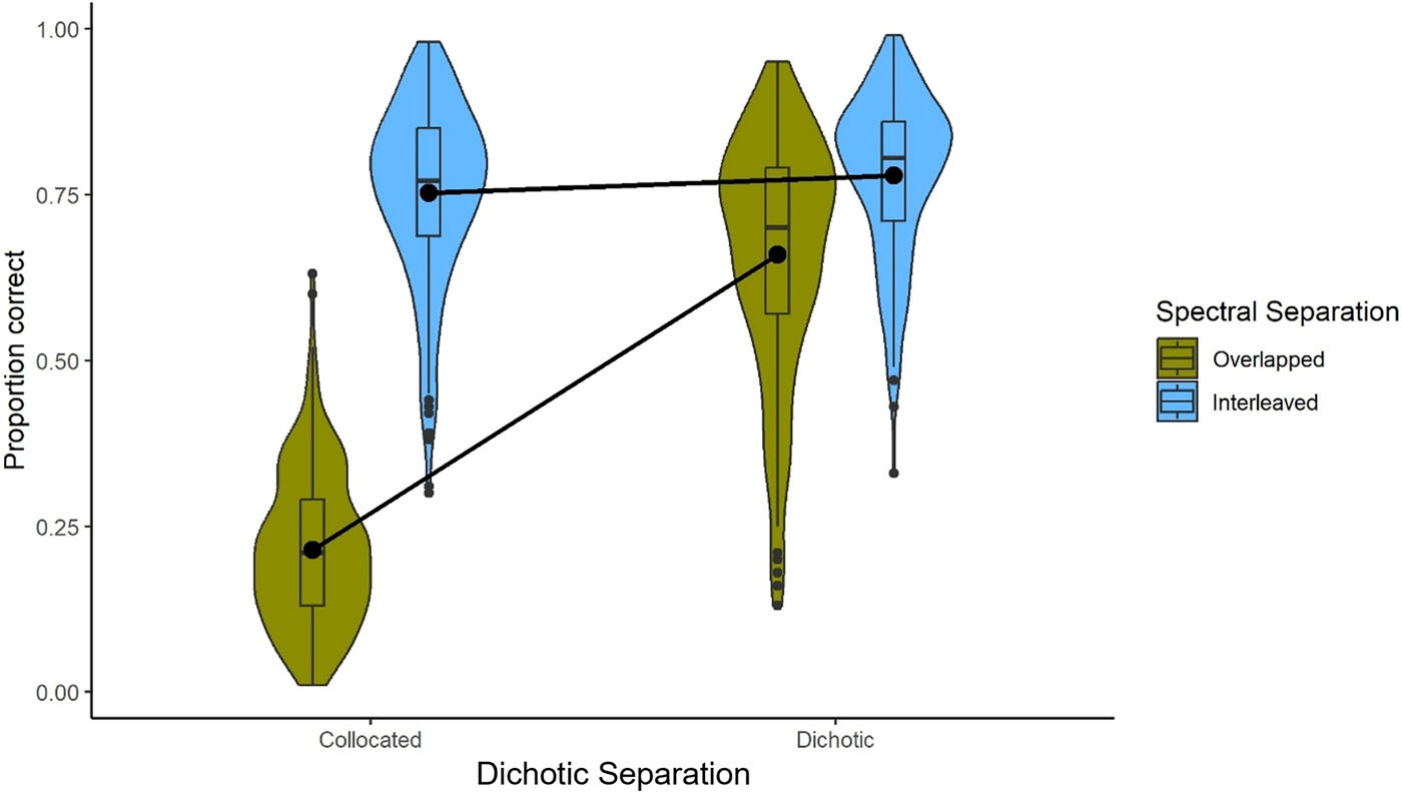
Average performance for each Dichotic Separation and Spectral Separation condition in the listening task.

Performance under each listening condition is shown in Fig. 2. The first GLMM included main effects and interactions of Dichotic Separation (collocated, dichotic) and Spectral Separation (overlapped, interleaved), along with six cognitive test scores. The model included random intercepts and slopes for Dichotic Separation and Spectral Separation at both the participant and item levels^1^. Performance for each participant on each trial of the listening task was expressed as a proportion between 0 and 1. The two levels of Dichotic Separation and Spectral Separation were sum‐coded as −1 and +1, respectively. The full model specification was as follows:^2^

$$\begin{array}{ccc} & & \mathrm{Proportion}\_\mathrm{Correct}∼\mathrm{DichoticSeparation}*\mathrm{Spectral}\mathrm{Separation}*\\ & & \left(\mathrm{Phon}\_\mathrm{NW}+\mathrm{Phon}\_\mathrm{RJ}+\mathrm{Att}\_\mathrm{RSVP}+\mathrm{Att}\_\mathrm{Singleton}+\mathrm{Exec}\_\mathrm{LNS}+\mathrm{Exec}\_\mathrm{RSpan}\right)\\ & & +\;\left(1+\mathrm{Dichotic}\mathrm{Separation}+\mathrm{Spectral}\mathrm{Separation}|\mathrm{participant}\right)+\left(1+\mathrm{DichoticSeparation}+\mathrm{SpectralSeparation}|\mathrm{item}\right) \end{array}$$

Using generalized variance inflation factors (GVIF; Dancey & Reidy, 2007), we determined that there were no concerns regarding multicollinearity amongst the predictors, including the six cognitive scores: all GVIF values were low (range: 1.00–1.35). Significance testing was conducted via likelihood ratio tests comparing the full model to reduced models. The main effect of Dichotic Separation was significant, χ^2^ (1) = 350.66, *p* < .001, revealing significantly worse performance in the collocated condition (*M* = 0.48, *SD* = 0.38) compared to the dichotic condition (*M* = 0.72, *SD* = 0.29). The main effect of Spectral Separation was also significant, χ^2^ (1) = 563.76, *p* < .001, revealing significantly worse performance in the overlapped condition (*M* = 0.44, *SD* = 0.36) compared to the interleaved condition (*M* = 0.77, *SD* = 0.27). The main effects of Phon_RJ (*χ* ^2^ (1) = 17.91, *p* < .001), Att_RSVP (*χ* ^2^ (1) = 5.79, *p* < .05), and Exec_LNS (*χ* ^2^ (1) = 6.57, *p* < .05) were significant, indicating that, overall, higher Phon_RJ, Att_RSVP and Exec_LNS scores were associated with higher accuracy scores on the listening task. There was also a significant interaction between Dichotic Separation and Spectral Separation, *χ* ^2^ (1) = 351.79, *p* < .001. This interaction is described in more detail in the second GLMM.

Bivariate Pearson correlation coefficients between cognitive tasks are reported in Table 2a. Most of the cognitive tasks were positively correlated with each other. The phonological tasks (Phon_NW and Phon_RJ) and the executive function tasks (Exec_LNS and Exec_RSpan) correlated with the highest number of other cognitive tasks, while the two attention tasks (Att_RSVP and Att_Singleton) did not correlate significantly with any other cognitive tasks.

**Table 2a:** Correlation matrix (Pearson's *r*) of all six cognitive tasks

	Non‐Word Repetition Task (Phon_ NW)	Rhyme Judgment Task (Phon_ RJ)	Rapid Serial Visual Presentation Task (Att_ RSVP)	Singleton Task (Att_ Singleton)	Letter‐Number Sequencing Task (Exec_ LNS)	Reading Span Task (Exec_ RSpan)
Phon_ NW	1.00	0.24	0.003	−0.03	0.32	0.24
Phon_ RJ	0.24	1.00	0.10	0.03	0.30	0.23
Att_ RSVP	0.003	0.10	1.00	−0.01	0.05	0.07
Att_ Singleton	−0.03	0.03	−0.01	1.00	0.11	0.06
Exec_ LNS	0.32	0.30	0.05	0.11	1.00	0.37
Exec_ RSpan	0.24	0.23	0.07	0.06	0.37	1.00

Cronbach's alpha scores for each cognitive task are reported in Table 2b. For the Att_RSVP task, two scores are reported: one for the selective attention condition and one for the divided attention condition. Three tasks—one from each domain—showed poor internal consistency (Phon_NW, Exec_LNS and Att_RSVP). However, the remaining three tasks produced good or acceptable scores, with one (Phon_RJ) demonstrating excellent consistency.

**Table 2b:** Internal consistency (Cronbach's alpha scores) of all six cognitive tasks

Phon_ NW	Phon_ RJ	Att_ RSVP (selective)	Att_ RSVP (divided)	Att_ Singleton	Exec_ LNS	Exec_ RSpan
0.38	0.95	0.37	0.45	0.81	0.55	0.73

For the second GLMM, the scores from the six cognitive tasks were entered into an exploratory factor analysis to derive a single underlying cognitive factor. The uniqueness and loading values of each cognitive task are reported in Table 3. Visual inspection of the scree plot and associated eigenvalues suggested that one factor was sufficient to represent the data. This was confirmed by a chi‐square test (*p* = .87), indicating that the single‐factor model represented the data well.

**Table 3:** Outputs of the factor analysis showing the uniqueness and loading values of each cognitive task

	Phon_NW	Phon_RJ	Att_RSVP	Att_Singleton	Exec_LNS	Exec_RSpan
Uniqueness	0.779	0.791	0.991	0.990	0.528	0.724
Loading	0.470	0.457	0.093	0.100	0.687	0.525

Individual factor scores were derived from this factor analysis to create a measure of overall cognitive ability for each participant (labeled “CogFact”). The CogFact scores were then incorporated into the second GLMM as an additional factor alongside Dichotic Separation and Spectral Separation. This GLMM included all main effects and all two‐way and three‐way interactions. As before, random intercepts and slopes for Dichotic Separation and Spectral Separation were modeled at the participant and item levels,^3^ and the levels of Spectral Separation and Dichotic Separation were sum‐coded as −1 and +1, respectively. The full three‐factor model was specified as

$$\begin{array}{ccc} & & \mathrm{Proportion}\_\mathrm{Correct}∼\mathrm{Dichotic}\mathrm{Separation}*\mathrm{Spectral}\mathrm{Separation}*\mathrm{CogFact}\\ & & +\;\left(1+\mathrm{Dichotic}\mathrm{Separation}+\mathrm{Spectral}\mathrm{Separation}|\mathrm{participant}\right)+\left(1+\mathrm{Dichotic}\mathrm{Separation}+\mathrm{Spectral}\mathrm{Separation}|\mathrm{item}\right). \end{array}$$

The second GLMM had a slightly lower Bayesian Information Criterion (BIC; 56,846), compared with the first GLMM (57,015), suggesting that the use of the CogFact scores produced a better fit for the data than using the six individual cognitive task scores.

**Fig. 3:**
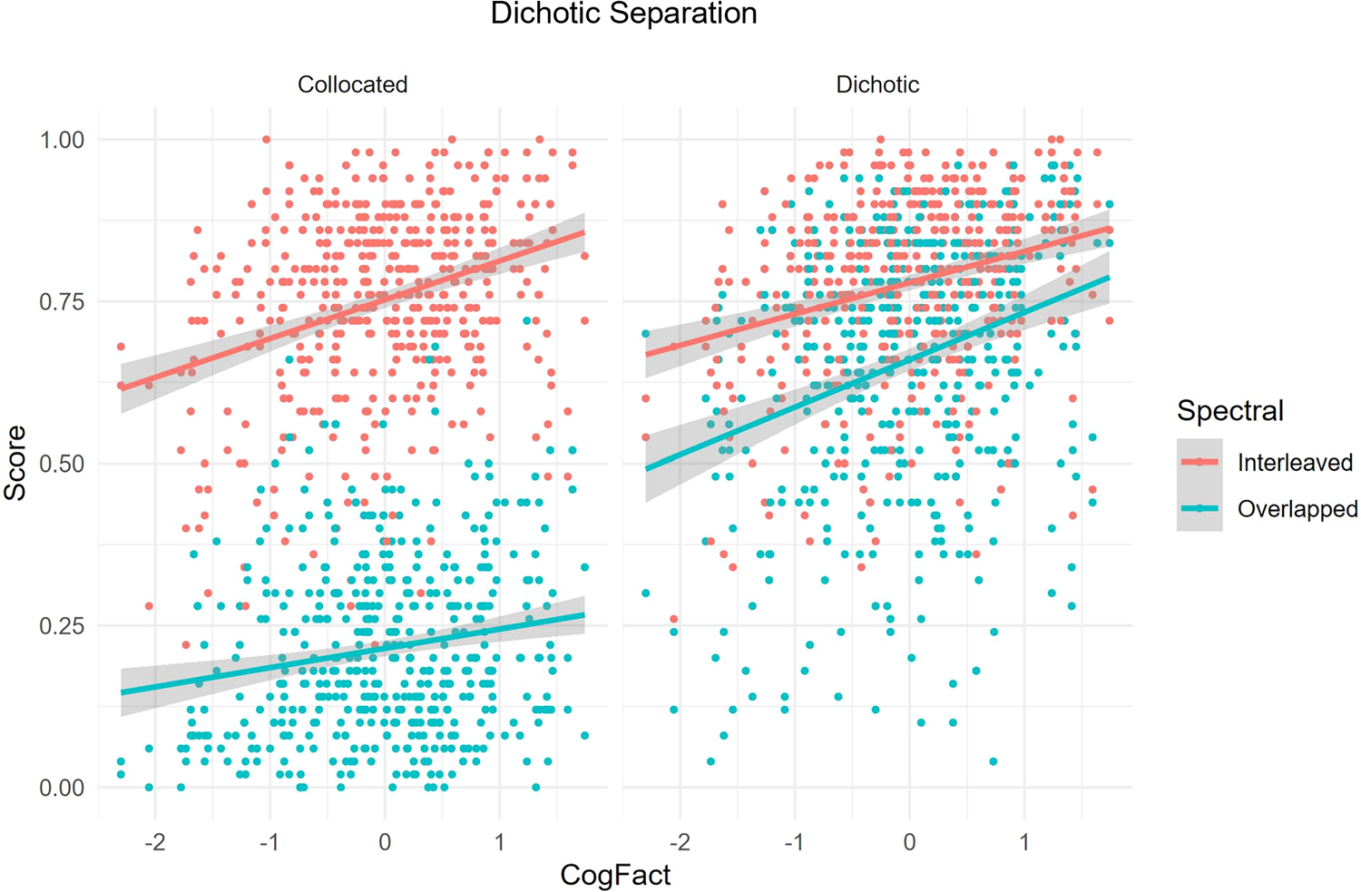
Listening performance (Proportion Correct) as a function of individual CogFact scores for each of the four conditions of the design. Note that the weakest relationship is seen in the Collocated_Overlapped condition.

The results of the second GLMM are presented in Table 4a. This analysis produced similar main effects and interaction of Dichotic Separation and Spectral Separation to the GLMM reported previously. In addition, there was a significant main effect of CogFact, *χ^2^* (1) = 35.59, *p* < .001, suggesting that high cognitive capacity was associated with superior listening performance. There were no significant two‐way interactions involving CogFact. However, a significant three‐way interaction between CogFact, Dichotic Separation, and Spectral Separation was obtained, *χ^2^* (1) = 11.32, *p* < .001. This interaction is illustrated in Fig. 3. Post hoc tests using the *emmeans()* function provided the standard error values of each listening condition (Collocated_Overlapped = 0.07, Collocated_Interleaved = 0.06, Dichotic_Overlapped = 0.07, Dichotic_Interleaved = 0.07). These values suggest that variability was similar across the four listening conditions, indicating that there were neither floor nor ceiling effects in any condition.

**Table 4a:** Results from Likelihood Ratio Tests conducted for the overall generalized linear mixed‐effects model with CogFact, Dichotic Separation, and Spectral Separation

	CogFact	Dichotic Separation	Spectral Separation	CogFact: Dichotic Separation	CogFact: Spectral Separation	Dichotic Separation: Spectral Separation	CogFact: Dichotic Separation: Spectral Separation
***χ^2^***	35.59	349.95	562.22	1.60	0.55	348.85	11.32
*p*‐value	<.001 ^***^	<.001 ^***^	<.001 ^***^	0.21	0.46	<.001 ^***^	<.001 ^***^

^*^

Post hoc pairwise comparisons were conducted using *emmeans* *()* to unpack the significant interaction between Dichotic Separation and Spectral Separation. The results are presented in Table 4b. All pairwise comparisons were significant (*p* < .05), but the estimates suggest that performance was poorest when there was neither dichotic nor spectral RM (Collocated_Overlapped) and best when there was both dichotic and spectral RM (Dichotic_Interleaved). Of the two interim conditions, performance was better when only spectral RM was present, compared to when only dichotic RM was present.

**Table 4b:** Post hoc comparisons of listening performance across pairs of listening conditions (Tukey‐corrected)

	C_I – D_I	C_I – C_O	D_I – D_O	C_O – D_O	C_I – D_O	D_I – C_O
Estimate	−0.18	2.82	0.69	−2.31	0.51	3.00
Standard error	0.07	0.07	0.07	0.08	0.08	0.08
*p‐*value	<.05 ^*^	<.001 ^***^	<.001 ^***^	<.001 ^***^	<.001 ^***^	<.001 ^***^

To assess how the relationship between CogFact and task performance varied across the four main listening conditions (i.e., the significant three‐way interaction), we tested estimated marginal trends (simple slopes) using the *emtrends()* function for all conditions and all possible pairwise comparisons. The results are presented in Table 4c. Inspection of the 95% confidence intervals for the estimated CogFact slopes indicated that all intervals excluded zero, suggesting that CogFact significantly predicted performance in each condition. The pairwise comparisons showed no significant differences between conditions, but the estimated slope of CogFact under Collocated_Overlapped was the smallest, reflecting a weaker positive relationship between CogFact and listening performance in the most challenging condition.

**Table 4c:** Post hoc tests of the relationship between CogFact and listening performance across listening conditions (Tukey‐corrected)

	C_I	C_O	D_I	D_O		
Estimate	0.31	0.23	0.30	0.34		
Standard error	0.06	0.06	0.06	0.06		
	C_I – D_I	C_I – C_O	D_I – D_O	C_O – D_O	C_I – D_O	D_I – C_O
Estimate	0.01	0.08	−0.03	−0.11	−0.02	0.07
Standard error	0.04	0.04	0.04	0.04	0.06	0.05
*p‐*value	.99	.14	.82	.05	.98	.39

*Note*. C = collocated, D = dichotic, O = overlapped, I = interleaved.

## 5 Discussion

Understanding speech in the presence of a competing talker can be challenging due to both EM (spectro‐temporal overlap between target and masker) and the need to stream, and selectively attend to, the voices. Speech‐on‐speech listening can be facilitated through separating the two talkers either spatially (via manipulating perceived location or ear of presentation) or spectrally (via filtering the voices into non‐overlapping frequency bands). Both types of separation reduce EM as well as providing cues to streaming. The performance benefits that this creates—known as spatial (dichotic) and spectral RM—are well documented; however, certain key questions remain unanswered. In particular, the relative benefit of these two types of RM, the effects of combining them, and the relationship of these benefits to individual differences in cognitive abilities are unclear. In the current study, we addressed these questions using a selective listening task, in which participants heard meaningful sentences spoken by two talkers simultaneously and were cued to report the sentence spoken by one of the talkers. The talkers were presented as either collocated (no dichotic RM) or dichotic (dichotic RM) and in spectrally overlapping (no spectral RM) or spectrally interleaved (spectral RM) frequency bands. We also administered a battery of cognitive tasks and used them to derive a single overall cognitive score for each participant. The results from the specific cognitive tests and the combined cognitive score were then used to assess the relationships between cognition, listening performance, and RM effects.

### 5.1 RM

Performance on the listening task was generally high (>60% correct), with the exception of the most challenging listening condition (Collocated_Overlapped) in which neither dichotic nor spectral RM was present, where performance dropped to 21%. As this pattern would suggest, we found main effects of both dichotic and spectral separation: Performance was higher in the dichotic than the collocated conditions and in the spectrally interleaved than the spectrally overlapping conditions. These results support a range of previous studies demonstrating that spatial (dichotic) and spectral separation can improve intelligibility of a target during speech‐on‐speech listening (e.g., Kidd et al., 2010; Knight et al., 2023; Litovsky, 2012).

Performance improvements were similar when only one (of either dichotic or spectral) RM was present: performance improved relative to the no‐RM condition (Collocated_Overlapped) by 45% when stimuli were dichotically (but not spectrally) separated and by 54% when stimuli were spectrally (but not dichotically) separated. This suggests that, at least in the context of the dichotic and spectral separation used in this study, listeners are able to exploit both cues to a similar extent—a finding in line with Best et al. (2006). However, we also found a significant interaction between dichotic and spectral separation, with post hoc tests revealing differences between all four listening conditions. Thus, despite the generally similar magnitude of RM effects, there was nevertheless a significant difference between the conditions with spectral RM only (Collocated_Interleaved) and dichotic RM only (Dichotic_Overlapped), with better performance seen in the spectral RM condition. This seems likely to reflect differences in processing at higher levels of the auditory pathway. Specifically, information from our two ears is integrated in the midbrain (e.g., Palmer, Shackleton, & McAlpine, 2002), potentially allowing for some cross‐ear interference higher in the auditory pathway despite dichotic release from EM at the cochlear level. However, information in different frequency bands is not integrated in this way, with tonotopy (i.e., the activation of distinct neural locations by different frequencies) being maintained through to the level of the auditory cortex (e.g., Humphries, Liebenthal, & Binder, 2010). As a result, there is less potential for cross‐frequency interference at these higher processing levels. From this perspective, then, it makes sense that a larger performance benefit might arise from spectral, rather than dichotic, RM.

This finding should not, however, be taken to indicate that spectral RM always provides the largest perceptual benefit; instead, it seems likely to be a product of the current experiment's somewhat artificial listening situation. In real‐world contexts, discrete spectral separation (as implemented here) is rare: spectra typically overlap, with spectral cues to streaming arising from moment‐by‐moment changes in the degree of overlap.^4^ In other words, data availability arising from real‐world spectral differences tends to be less extensive and more dynamic than in our experiment. Furthermore, in the context of common real‐world spectral differences, such as those related to voice differences (e.g., male vs. female voices), it may be that listeners rely more heavily on spatial than spectral cues (Allen et al., 2008). That is, listeners in real‐world situations may weigh spectral cues less heavily for a variety of reasons. Finally, any comparisons of the relative benefit of dichotic and spectral RM need to be contextualized within a range of possible performance benefits. In other words, although the overall magnitude of dichotic versus spectral RM appeared to be similar in the current study, dichotic presentation represents the upper limit of RM (no further separation is possible), whereas spectral RM may afford much larger benefits than observed here if implemented in a different spectro‐temporal configuration. Such questions remain to be investigated.

Despite the slightly larger performance benefit derived from spectral RM relative to dichotic RM, it was not the case that spectral RM rendered dichotic RM ineffective: We observed a significant additional improvement in performance when both types of RM were present, compared to either type of RM alone. The reasons for this are unclear. However, one possibility is that the addition of dichotic RM on top of spectral RM caused a perceptual reweighting of the existing spectral cues in a manner that was beneficial to performance. Regardless of the underlying mechanism, however, it is clear that the highest performance occurred when both types of RM were present. This is in line with Best et al. (2013). It is also supported by the work of Ihlefeld and Shinn‐Cunningham (2008), who found that both timbre and location cues contributed independently to listeners’ ability to accurately report a target sentence during speech‐on‐speech listening, with performance highest when both were present as opposed to either feature alone.

The observed interaction between dichotic and spectral separation reflects, in part, the fact that only a relatively small (albeit significant) additional benefit arises when both types of RM are present, compared to either spectral or dichotic RM alone (see Fig. 2). One possibility is that spectral and dichotic RM in fact combine additively, and that the observed interaction results from ceiling effects. However, it should be noted that the mean accuracy in the easiest condition was only around 75% correct, leaving room for improvement. Nevertheless, future studies should attempt to address this question by targeting lower overall performance levels.

Finally, it must be remembered that dichotic listening was used in the current study as a proxy for true spatial hearing. The generalizability of the current findings to situations involving cues such as ITDs and free‐field presentation of sound sources remains to be established.

### 5.2 Contribution of cognitive abilities to listening performance

Scores on our combined cognitive factor showed a positive relationship with overall listening performance. This is in line with the prevailing view that good cognition supports successful speech‐in‐noise listening (e.g., Akeroyd, 2008; Rönnberg et al., 2022). However, there was also a highly significant three‐way interaction between dichotic separation, spectral separation, and cognition. Post hoc analyses revealed that, although still positive, the weakest relationship between performance and cognition was observed for the most challenging listening condition, that is, the one in which neither dichotic nor spectral RM was present (Collocated_Overlapped). Although overall performance was poorest in this condition, variability was comparable to that in the other conditions—in other words, the weaker relationship seems unlikely to be due to a floor effect. Furthermore, this correlation was weaker than the correlation between cognition and listening performance in either of the conditions featuring only one type of RM. Although these post hoc comparisons did not reach significance after correction for multiple tests, their broad pattern supports an account of speech‐on‐speech listening in which the relative balance between data and resource limits shifts according to the nature of the stimuli. As described above, when EM is strong and/or cues to streaming are absent, a data limit is reached, and there is no longer enough of the signal available for the application of cognitive resources to have a strong impact on performance (cf., Norman & Bobrow, 1975). This interpretation is in line with Mattys et al.’s (2025) DRL framework and adds nuance to models such as the ease of language understanding model (Rönnberg et al., 2022), which suggest that cognitive resources are always positively linked to performance during listening in challenging conditions, regardless of the nature or extent of the challenge. A data‐ and resource‐limit account would impose a boundary on the extent of this positive relationship.

The main effects of Phon_RJ, Att_RSVP and Exec_LNS in our first GLMM showed that, of our six cognitive tasks, one measure of phonological processing (Rhyme Judgment), one measure of attention (RSVP), and one measure of executive function (LNS) were associated with performance across all conditions of the listening task. This finding is in line with existing research highlighting the importance of phonological skills, executive function and domain‐general selective attention when listening to speech in noisy environments (Clayton et al., 2016; Millman & Mattys, 2017; Smith & Pichora‐Fuller, 2015). However, it must be noted that the internal consistency of both the Att_RSVP and Exec_LNS measures was low. On the one hand, low consistency may lead to an underestimation of the true relationship between variables; on the other hand, it may mean that any identified relationships are potentially unreliable. By contrast, the Phon_RJ measure had excellent internal consistency. As a result, only a relationship between phonological processing and listening performance can be asserted with confidence.

Interestingly, the other measure of executive function—the RSpan—was not associated with listening performance, despite its acceptable internal consistency. This is surprising given that span tasks of this nature are often considered a “gold standard” measure of cognition in the speech‐in‐noise literature, and are thought to capture a range of complex WM skills relevant for successful speech perception in challenging environments (Rönnberg et al., 2022). Although a relationship is not always observed between speech‐in‐noise listening and RSpan scores, particularly in young normal‐hearing adults, this is suggested to be because of a lack of a detectable relationship between speech perception and cognition in general for this group (Fullgräbe & Rosen, 2016) and not—as is the case here—because other cognitive measures provide greater predictive power. One factor to consider is that Reading Span Tasks, and indeed span tasks in general, exist in diverse formats and can be implemented in a variety of ways. Although the underlying mechanism is not yet well understood, different implementations of the RSpan not only produce different patterns of performance but also appear to have different relationships to speech‐in‐noise perception (Heinrich, 2025). The absence of a relationship between the RSpan and listening performance in the current study may therefore reflect the specific details of our implementation in addition to the role of complex WM and executive function in speech‐on‐speech listening in general.

BIC values indicated that the GLMM fitted with the combined cognitive factor (CogFact) was a slightly better fit to the data than the GLMM that used all six cognitive tasks separately. This suggests that combining the cognitive task scores into a single factor captured a meaningful underlying general ability that could be related to variation in performance on the listening task. Nevertheless, it is worth noting that the two tests of attention (Singleton and RSVP tasks) did not correlate with any of the other cognitive measures and produced low loadings on our combined cognitive factor. It is possible that, although our tasks were developed from existing paradigms (e.g., Adamo, Pun, Pratt, & Ferber, 2008; Theeuwes, 1992), task‐specific demands rendered the data too noisy to reliably detect individual differences in attentional control. The fact that we observed a significant relationship between individual RSVP scores and listening task performance may at first suggest that this explanation is unlikely. Instead, it may be tempting to conclude that attention is separable from other cognitive domains such as executive function and phonological processing. However, given the substantial body of work linking attention to other facets of cognition, this also seems unlikely (e.g., Baddeley, 2000; Diamond, 2013). Furthermore, the poor internal consistency for the RSVP task makes any relationships between it and other measures questionable. An alternative possibility is that shared variability in the attention tasks and listening tasks may have been introduced by the online nature of the experiment and the resulting difficulty in controlling for potentially important hardware‐related factors such as screen size, visual angle and contrast (Chen, Liao, & Yeh, 2011; Yazgan, Yağımlı, & Ozubko, 2024) or participant‐related factors such as attention and focus during time‐sensitive tasks. Future studies could investigate the degree of variability in online task performance due to these factors and the possibility of measuring and/or controlling them remotely.

Finally, we observed a high correlation between dichotic and spectral RM effects. In other words, participants who accrued a large performance benefit from one type of RM tended to also benefit from the other type. This suggests that broadly similar underlying skills are required to exploit the additional information made available through RM. This is despite the fact that the cues involved are of a very different nature, with dichotic release involving manipulation of the locus of sound presentation and spectral release involving manipulation of the sound's physical properties. Furthermore, there is likely to be a considerable range of individual differences in cue use and strategy during speech‐in‐noise listening. With these factors in mind, it is plausible that the ability to exploit dichotic versus spectral RM might vary within an individual. Nevertheless, the results reported here are in line with existing work indicating that an individual's speech‐on‐speech listening ability is correlated not just across different types of masking but for similar masking tasks across different modalities (Byrne, Conroy, & Kidd, 2023).

## 6 Conclusion

In this study, we explored the effects of spatial (specifically dichotic) and spectral RM during speech‐on‐speech listening using a selective attention paradigm in which participants had to attend to a target voice which was separated from the masker voice either dichotically, spectrally, both, or not at all. We observed significant performance benefits for both types of RM, with the best performance shown when both were present simultaneously. We also administered a battery of cognitive tasks in order to assess the relationship of these RM benefits to individual differences in cognitive abilities. The results revealed that the relationship between cognitive ability and listening performance was generally positive but weakest for the most challenging listening condition (i.e., the condition in which no RM was present). Short‐term phonological memory, executive function, and attention seemed to be the strongest predictors of absolute listening performance. We additionally observed a strong correlation between dichotic and spectral RM benefits, suggesting that broadly similar underlying skills are required to exploit RM cues. We interpret these results in terms of a data‐ and resource‐limit framework, which we suggest may be a useful tool to account for speech perception performance across a range of different tasks and listening environments. Future use of the framework may allow us to add nuance to existing theories of the relationship between speech perception and cognitive abilities.

## Notes

1 More complex models with any additional slopes of Dichotic Separation:Spectral Separation for participant and item intercepts failed to converge.

2 This model produced a warning message – “Model is nearly unidentifiable: large eigenvalue ratio”. A simpler model setting the number of Gauss‐Hermite quadrature points (nAGQ) to 0 converged without error and produced very similar results to the model reported here.

3 More complex models with any additional slopes of Dichotic Separation:Spectral Separation for participant and item intercepts failed to converge.

4 Complete dichotic isolation of sound sources, as implemented here, is also rare; however, it seems likely that listeners are more familiar with the process of attending to different spatial locations (even when signalled entirely by ear of presentation) than with attending to separate spectral regions.
